# Relationship between IgE Levels Specific for Ragweed Pollen Extract, Amb a 1 and Cross-Reactive Allergen Molecules

**DOI:** 10.3390/ijms24044040

**Published:** 2023-02-17

**Authors:** Lauriana-Eunice Zbîrcea, Maria-Roxana Buzan, Manuela Grijincu, Elijahu Babaev, Frank Stolz, Rudolf Valenta, Virgil Păunescu, Carmen Panaitescu, Kuan-Wei Chen

**Affiliations:** 1 Center of Immuno-Physiology and Biotechnologies, Department of Functional Sciences, Victor Babes University of Medicine and Pharmacy, 300041 Timisoara, Romania; 2 OncoGen Center, Pius Brinzeu County Clinical Emergency Hospital, 300723 Timisoara, Romania; 3 Vienna Competence Center, Biomay AG, 1090 Vienna, Austria; 4 Center of Pathophysiology, Infectiology and Immunology, Department of Pathophysiology and Allergy Research, Division of Immunopathology, Medical University of Vienna, 1090 Vienna, Austria; 5 Laboratory for Immunopathology, Department of Clinical Immunology and Allergology, Sechenov First Moscow State Medical University, 119991 Moscow, Russia; 6 Karl Landsteiner University of Health Sciences, 3500 Krems, Austria; 7 NRC Institute of Immunology FMBA of Russia, 115522 Moscow, Russia

**Keywords:** allergy, allergen, molecular diagnosis, ragweed pollen allergy, specific IgE, cross-reactivity, profilin, calcium-binding allergens, cross-reactive carbohydrate determinants

## Abstract

Ragweed (*Ambrosia artemisiifolia*) pollen is a major endemic allergen source responsible for severe allergic manifestations in IgE-sensitized allergic patients. It contains the major allergen Amb a 1 and cross-reactive allergen molecules, such as the cytoskeletal protein profilin, Amb a 8 and calcium-binding allergens Amb a 9 and Amb a 10. To assess the importance of Amb a 1, profilin and calcium-binding allergen, the IgE reactivity profiles of clinically well-characterized 150 ragweed pollen-allergic patients were analysed regarding specific IgE levels for Amb a 1 and cross-reactive allergen molecules by quantitative ImmunoCAP measurements, IgE ELISA and by basophil activation experiments. By quantifying allergen-specific IgE levels we found that Amb a 1-specific IgE levels accounted for more than 50% of ragweed pollen-specific IgE in the majority of ragweed pollen-allergic patients. However, approximately 20% of patients were sensitized to profilin and the calcium-binding allergens, Amb a 9 and Amb a 10, respectively. As shown by IgE inhibition experiments, Amb a 8 showed extensive cross-reactivity with profilins from birch (Bet v 2), timothy grass (Phl p 12) and mugwort pollen (Art v 4) and was identified as a highly allergenic molecule by basophil activation testing. Our study indicates that molecular diagnosis performed by the quantification of specific IgE to Amb a 1, Amb a 8, Amb a 9 and Amb a 10 is useful to diagnose genuine sensitization to ragweed pollen and to identify patients who are sensitized to highly cross-reactive allergen molecules present in pollen from unrelated plants, in order to enable precision medicine-based approaches for the treatment and prevention of pollen allergy in areas with complex pollen sensitization.

## 1. Introduction

Immunoglobulin E-associated allergy is considered the most common immune disorder, affecting almost 30% of the population worldwide [1]. *Ambrosia artemisiifolia* (common or short ragweed) originated in North America [2] and is an important allergen source now distributed worldwide [3,4,5,6,7]. The endemic dispersal of ragweed throughout Europe is increasing [8] due to environmental changes such as urbanization and climate change, having a great impact on public health [9,10,11]. Ragweed pollen is a major cause of allergic rhinitis in late summer, inducing asthma about twice as often as other types of pollen [12].

Currently, the most common procedure for allergy diagnosis is skin prick testing (SPT) using allergen extracts [13,14]. Alternatively, serological IgE tests mainly based on extracts are performed [15,16]. The drawback of extract-based tests is the allergen content heterogenicity, which may lead to the underrepresentation of certain allergens with potential wrong diagnosis results as consequences [14,17]. Therefore, component-resolved diagnostics (CRD) [18,19] have been proposed as an alternative for more accurate diagnosis. In ragweed pollen, eleven proteins were identified as allergens and included in the International Union of Immunological Societies (IUIS) database [20]. However, currently only the major ragweed allergen Amb a 1, and Amb a 4, an allergen which is structurally related to the major mugwort pollen allergen, Art v 1, a defensin-like protein [21], are available for CRD in singleplex [22] or multiplex systems [23,24]. Testing with Amb a 1 and Amb a 4, as well as with Art v 1 and Art v 6, the Amb a 1-related allergen from mugwort, is useful to discriminate between genuine sensitization to ragweed and mugwort pollen.

However, among the eleven ragweed allergens, there are molecules such as Amb a 8, belonging to the profilin family, and Amb a 9 and Amb a 10, belonging to the calcium-binding protein (CBP) family [25]. These two protein families can be found in various allergen sources and are also known as pan-allergens [26]. Pan-allergens within an allergen source represent a challenge in allergy diagnosis due to their ability to cross-react with their homologues from other sources, thus obscuring the genuinely sensitizing allergen source [27]. Furthermore, relatively little is known about the contribution of pan-allergen IgE sensitization to clinical symptoms and their role in allergen-specific immunotherapy (AIT). Therefore, intensive studies on cross-reactivity and clinical relevance are needed before the decision to include them in CRD is made. Other cross-reactive components that may give false positive results are cross-reactive carbohydrate determinants (CCD), which do not seem to have relevant allergenic activity but are found in multiple allergen sources [28,29].

This study aimed to improve ragweed pollen allergy diagnosis by assessing the IgE reactivity and possible clinical relevance of ragweed pan-allergens Amb a 8, Amb a 9 and Amb a 10. Therefore, genuine sensitization towards ragweed and other common pollen allergen sources in western Romania, such as mugwort, birch and timothy grass [30], was evaluated in a clinically well-characterized and representative population of ragweed pollen allergic patients using marker allergens. Cross-reactivity was determined by measuring IgE binding towards profilins from the aforementioned allergens sources, and the allergenicity of these allergens was then evaluated in mediator release assays. The results of our study should answer the question of whether to include ragweed profilin, polcalcins and CCDs in ragweed allergy diagnosis to help allergists in guiding personalized forms of allergy treatment such as allergen-specific immunotherapy treatment (AIT).

## 2. Results

### 2.1. Clinical Characterization of the Ragweed-Allergic Patients Included in This Study

The study cohort comprises 150 ragweed-allergic patients (100 males and 50 females) aged between 18 and 61 years. All included patients reported rhinitis symptoms, 93% complained of conjunctivitis, 63% of asthma-like symptoms and 26% of skin symptoms upon ragweed pollen exposure. Rhinitis was defined based on symptoms such as nasal obstruction, rhinorrhoea, nasal pruritus and sneezing; conjunctivitis was defined based on tearing, ocular pruritus and ocular irritation symptoms. Asthma-like symptoms included dry cough, chest constriction, dyspnoea, and/or wheezing and skin symptoms included rash, pruritus and/or dryness. Sensitization to other common allergen sources was assessed by clinical history, positive SPT and/or serological IgE tests using allergen extracts. The prevalence of symptoms reported upon ragweed pollen exposure and other sensitizations are displayed in Table 1.

The majority (71%) of our study population is also sensitized to other allergen sources. The most prevalent other allergen sources are house dust mites (HDM) (37%), followed by grass pollen (34%), mugwort pollen (30%), tree pollen (20%), fungi (19%), dog (15%) and cat (15%) (Table 1).

### 2.2. Quantification of IgE Specific for Ragweed, CCD, Amb a 1 and Major Allergens from Pollens of Other Plants

**Figure 1:**
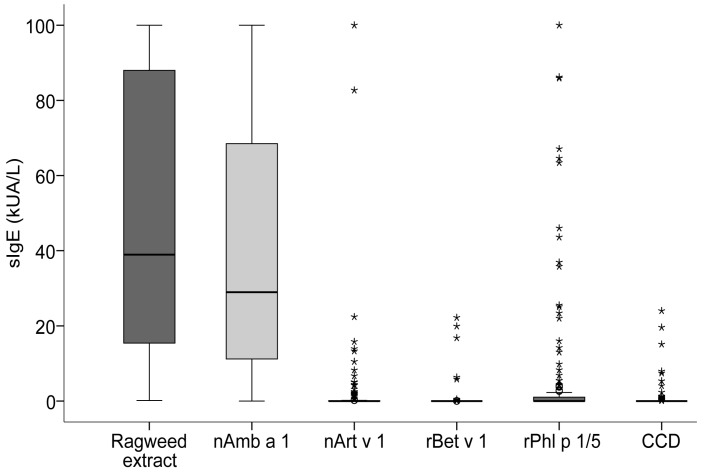
Box plot representation of specific IgE levels tested in ImmunoCAP (median = horizontal bars, boxes = interquartile range (IQR), whiskers = minimum and maximum not exceeding IQR; asterisks represent outliers exceeding 3.0 x IQR) against ragweed and other relevant pollen allergens (x-axis) in the study cohort (*n* = 150). Results are expressed as kUA/L (y-axis).

Table S1 shows the specific IgE (sIgE) levels against ragweed pollen extract, nAmb a 1, nArt v 1, rBet v 1, rPhl p 1/5b and CCD for 150 patients included in our study cohort and displayed in Figure 1. SIgE values ≥ 0.35 kUA/L were considered positive. IgE sensitization to ragweed extract was detected in 98.7% of the patients (*n* = 148), with positive IgE values ranging between 0.91 and 100 kUA/L (median 39.75). If one considered sIgE ≥ 0.1 kUA/L positive, all 150 patients can be considered IgE-positive to ragweed pollen extract. In total, 97.3% of the patients (*n* = 146) reacted to nAmb a 1, with sIgE values between 0.39 and 100 kUA/L (median 29.75). A total of 29.3% of the patients (*n* = 44) reacted to rPhl p 1/5b, with values between 0.35 and 100 (median 7.56), followed by 18.7% (*n* = 28) to nArt v 1 with values between 0.47 and 100 kUA/L (median 2.84) and 4% (*n* = 6) to rBet v 1 with values between 0.49 and 22.2 kUA/L (median 11.57). CCD-specific IgE was found in 13% of patients (*n* = 20) with values between 0.35 and 24 kUA/L (median 1.20) (Table S1).

### 2.3. IgE-Specific for nAmb a 1 Accounts for More than 50% of Ragweed Pollen sIgE in the Majority of Ragweed Pollen Allergic Patients

**Figure 2:**
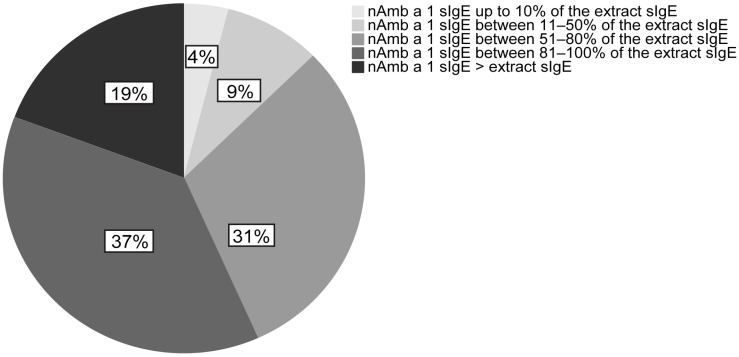
The percentage of patients showing different nAmb a 1 sIgE levels corresponding to ragweed extract IgE levels in 150 patients. nAmb a 1 sIgE value (kUA/L) was divided to extract sIgE to obtain the percentage for each patient. Based on the results obtained, patients were divided in 5 groups: nAmb a 1 sIgE made up to 10% of the extract sIgE, nAmb a 1 between 11 and 50%, nAmb a 1 between 51 and 80%, nAmb a 1 between 81 and 100%, and nAmb a 1 higher than extract.

Different levels of sIgE towards nAmb a 1 in the extract are displayed in Figure 2. In this analysis, 19% of the patients (*n* = 29) had more nAmb a 1 sIgE compared to extract sIgE, 37% of the patients (*n* = 56) had nAmb a 1 sIgE between 81 and 100%, 31% of the patients (*n* = 46) had nAmb a 1 sIgE between 51 and 80%, 9% of the patients (*n* = 13) had nAmb a 1 sIgE between 11 and 50% and 4% patients (*n* = 6) had sIgE against nAmb a 1 up to 10%, compared to extract sIgE.

### 2.4. The Role of Amb a 8 in Profilin Cross-Reactivity and Its Clinical Relevance

#### 2.4.1. Amb a 8 Is a Relevant Allergen According to Allergen-Specific IgE Levels and Frequency of Recognition

IgE sensitization to rAmb a 8 was detected in 21.3% (*n* = 32) of the patients with positive IgE values between 0.36 and 78.4 kUA/L (median 2.76) (Table 2). Sera positive to rAmb a 8 were also tested for IgE reactivity to other profilins: rArt v 4, rBet v 2 and rPhl p 12 in ImmunoCAP. All sera positive to rAmb a 8 were also positive to rArt v 4 and rPhl p 12, with IgE levels of 0.38–43.2 kUA/L (median 2.40) and 0.35–55.4 kUA/L (median 3.10), respectively, whereas 91% of the rAmb a 8 positive sera showed IgE reactivity to rBet v 2 with IgE levels of 0.05–41.2 kUA/L (median 2.29) (Table 2).

Statistical analysis showed that IgE levels of patients positive to rAmb a 8 are significantly higher compared to rPhl p 12 (Z = −3.852, *p* < 0.001) but lower compared to rArt v 4 (Z = −2.468, *p* < 0.05), while no statistical difference was found when compared to rBet v 2 (*p* > 0.05). However, our results indicate that out of nineteen patients with higher Art v 4-specific IgE, only five patients are sensitized to Art v 1.

#### 2.4.2. rAmb a 8 Binds the Majority of Profilin-Specific IgE as Determined by ImmunoCAP IgE Inhibition Experiments

IgE cross-reactivity between rAmb a 8, rArt v 4, rBet v 2 and rPhl p 12 was investigated by IgE inhibition experiments in ImmunoCAP using sera from patients which showed IgE reactivity to all four profilins (*n* = 29). Specific IgE levels obtained in ImmunoCAP after sera incubation with rAmb a 8 showed an IgE auto-inhibition of 81–100%. Specific IgE levels after the inhibition of the sera with rAmb a 8 showed an inhibition ranging from 71 to 100% of rBet v 2 sIgE, 82 to 99% inhibition of rPhl p 12 and 87 to 100% inhibition of rArt v 4 (Table 3). The inhibition of the sera with rArt v 4 showed an inhibition ranging from 59 to 97% of Amb a 8 sIgE.

#### 2.4.3. Basophil Activation Experiment Indicates the Relevant Allergenic Activity of Amb a 8

**Figure 3:**
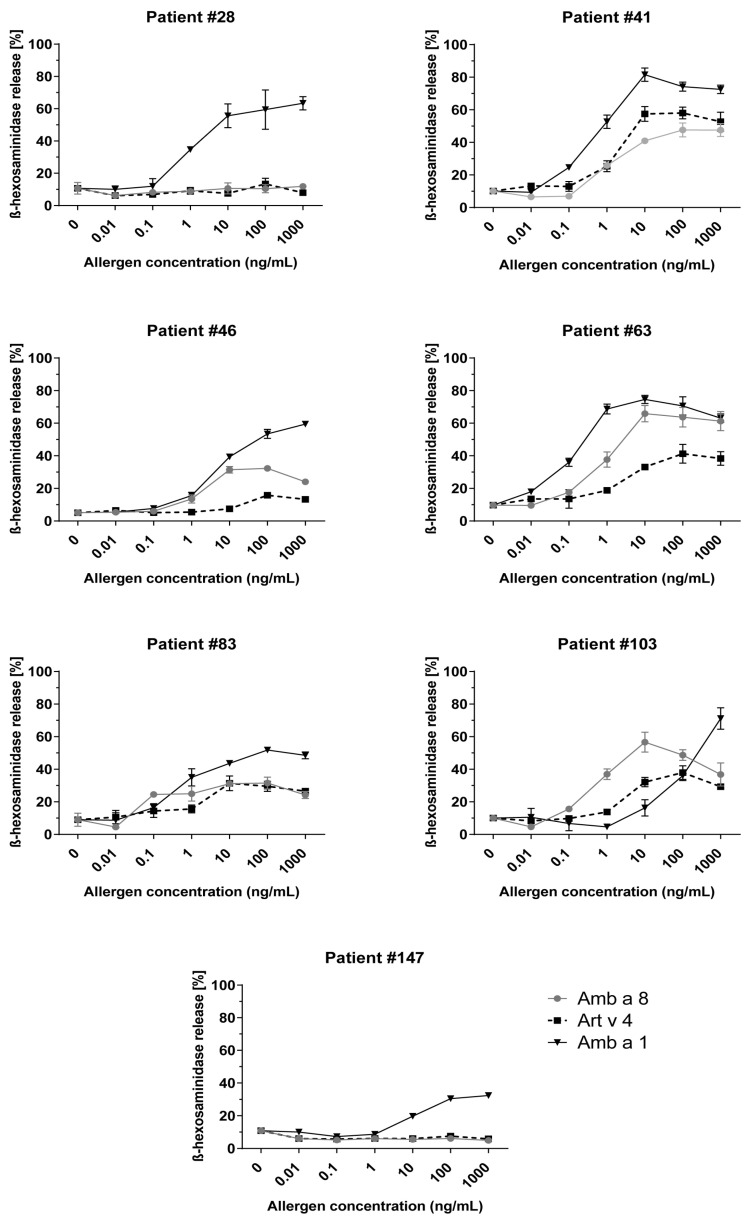
Allergenic activity of ragweed and mugwort profilins. RBL cells were loaded with sera of seven patients and stimulated with different concentrations of rAmb a 8 and rArt v 4; x-axes. β-hexosaminidase releases are expressed as percentages of total mediator contents +/− SD (y-axes). The allergenic activity of nAmb a 1 was evaluated in all patients as a reference.

The allergenic activity of rAmb a 8, rArt v 4 was determined by RBL cell mediator-release assay with seven sera from profilin-sensitized patients. Amb a 1 was used as the positive control. Maximal β-hexosaminidase release dose-dependent in these seven sera ranged between 6 and 65% for rAmb a 8, 7 and 58% for rArt v 4 and 32 and 81% for nAmb a 1 (concentration 10–1000 ng/mL), as shown in Figure 3.

Natural Amb a 1 induced the highest ß-hexosaminidase release of RBL cells in all patients, while five patients showed significant ß-hexosaminidase release when stimulated with different concentrations of rAmb a 8. rArt v 4 induced ß-hexosaminidase release in four patients upon stimulation with different concentrations of allergen. In comparison with rArt v 4, rAmb a 8 was able to trigger higher or similar degranulation of RBL cells for most of the patients, while rArt v 4 was able to induce a higher response in only one patient. These results were consistent with ImmunoCAP data showing that, in patients with high allergen-specific IgE levels, profilins had considerable allergenic activity.

### 2.5. Detection of IgE Specific for Calcium-Binding Allergens by ELISA

**Figure 4:**
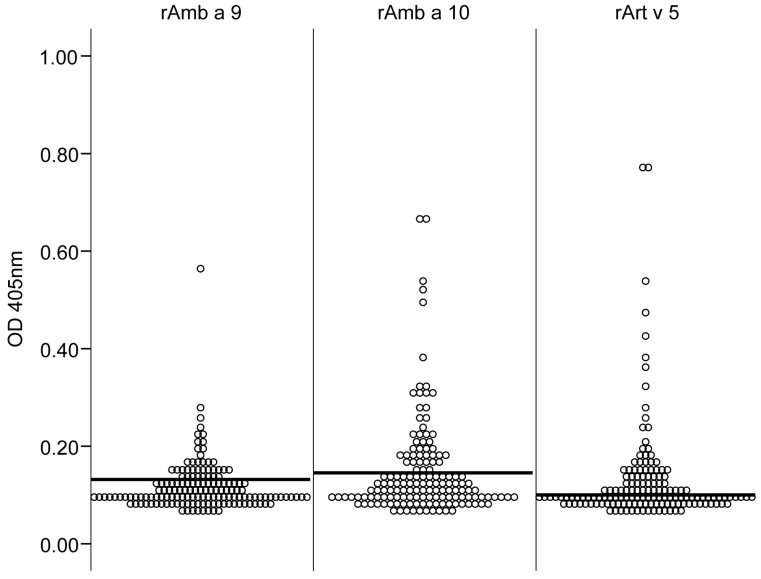
IgE reactivity to calcium-binding proteins rAmb a 9, rAmb a 10 and rArt v 5 was determined in ELISA assays using sera from ragweed-allergic patients (*n* = 150). The optical density values (OD 405 nm) displayed on the y-axis correspond to the levels of IgE antibodies. The cut-off value is indicated for Amb a 9 (0.1317), Amb a 10 (0.1453) and Art v 5 (0.100) allergens (horizontal line).

IgE reactivity to the calcium-binding allergens from ragweed and mugwort, rAmb a 9, rAmb a 10 and rArt v 5 was evaluated by ELISA (Figure 4). A total of 16% of the sera reacted with rAmb a 9 (*n* = 24), 25% reacted with rAmb a 10 (*n* = 38) and 35% reacted with rArt v 5 (*n* = 52). The IgE reactivity of each patient to these CBPs is included in Table S1.

### 2.6. Association of Clinical Symptoms with IgE Response to Ragweed Extract, nAmb a 1, Amb a 8, Amb a 9 and Amb a 10

**Figure 5:**
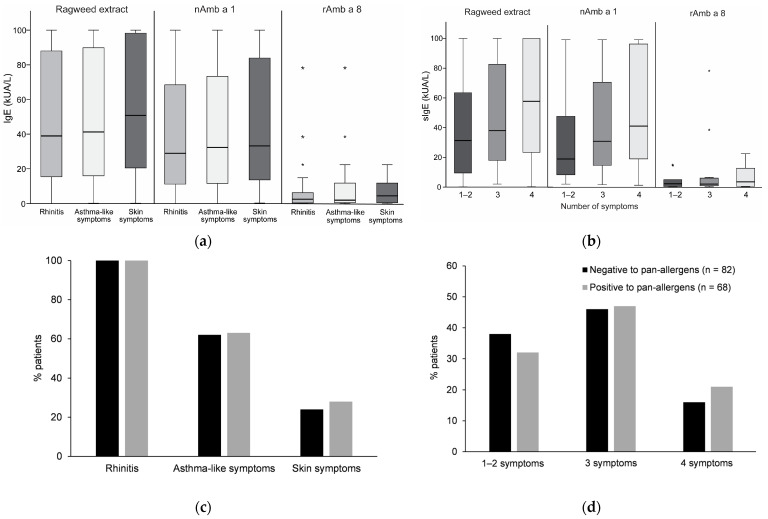
Association of the IgE response with clinical symptoms: patients show specific IgE levels to ragweed extract, Amb a 1 and Amb a 8 (x-axis) in relation to (**a**) prevalence of different symptoms and (**b**) the number of symptoms. Results are expressed as kUA/L (y-axis) (median = horizontal bars, boxes = IQR, whiskers = minimum and maximum not exceeding IQR; asterisks represent outliers exceeding 3.0 x IQR). (**c**) Prevalence of different symptoms and (**d**) number of symptoms (x-axis) in relation to reactivity towards any of the pan-allergens Amb a 8, Amb a 9 and Amb a 10 was expressed as percentage of patients (y-axis).

IgE levels of patients sensitized to ragweed extract, nAmb a 1 and Amb a 8 reporting different allergy symptoms, i.e., rhinitis, asthma-like symptoms and skin symptoms (Figure 5a), and a different number of symptoms—one or two, three and four (Figure 5b)—during the ragweed pollen season were displayed. We found that IgE levels to ragweed extract, nAmb a 1 or rAmb a 8 did not differ significantly between patients reporting different symptoms, nor in the patients reporting more symptoms (three and four symptoms) compared to those reporting fewer symptoms (one or two). However, there was a trend that patients with more than one to two symptoms had higher levels of specific IgE to ragweed pollen extract, nAmb a 1 and rAmb a 8 (Figure 5b).

Next, we verified whether there is a difference in the prevalence and number of different symptoms in patients positive to either rAmb a 8, rAmb a 9 or rAmb a 10 (*n* = 68) and patients negative to pan-allergens (*n* = 82), respectively (Figure 5b,c). Although we found no significant differences between the pan-allergen positive and negative patients regarding the prevalence and the number of symptoms, a higher proportion of pan-allergen positive patients reported four symptoms compared to pan-allergen negative patients (21% vs. 16%) (Figure 5c).

## 3. Discussion

The prerequisite for an accurate allergy diagnosis is the inclusion of relevant allergen molecules from the allergen source of interest in the diagnostic test, with the goal to obtain refined diagnostic information for the improvement of personalized treatment and prevention. In this study, we had the chance to study molecular IgE reactivity profiles to ragweed pollen allergen molecules in an area (western Romania) where ragweed is an endemic allergen source, similar to certain areas of the USA and the Lyon area in France [31,32]. Besides ragweed allergy, other common causes of seasonal respiratory allergies in this area are due to mugwort, birch and timothy grass pollen exposure. Previous studies conducted in western Romania showed that mugwort (*Artemisia vulgaris*), birch (*Betula sp.*) and timothy grass (*Phleum pratense*) are among the 20 most common allergen sources in this area, with high counts of pollen detected in spring, late summer and fall [30,33].

Antigen E, now termed major ragweed pollen allergen Amb a 1, was considered to be the most important ragweed pollen allergen and to be sufficient for allergen-specific immunotherapy of allergy to ragweed pollen [34]. However, more recent studies conducted in western Romania [35,36] investigating the sensitization profiles of ragweed-allergic patients showed complex IgE reactivity profiles. These studies suggest that besides Amb a 1, other allergens from ragweed, including pan-allergens, may play a role in ragweed sensitization and, therefore, should be considered for diagnosis and immunotherapy. In this study, we have investigated the importance of ragweed pan-allergens rAmb a 8 and rAmb a 9, as well as rAmb a 10 and the ability of Amb a 8 to cross-react to other common airborne allergens in the region, such as mugwort, birch and grass. Further, we also investigated IgE sensitization to CCDs in ragweed allergy diagnosis.

Using a large and clinically well-characterized population of ragweed pollen allergic patients, we started testing patients’ IgE reactivity towards ragweed extract and major allergens from ragweed, mugwort, birch and grass, nAmb a 1, nArt v 1, rBet v 1 and rPhl p 1/5b by ImmunoCAP (Figure 1). We found that almost all ragweed pollen-allergic patients were IgE sensitized to Amb a 1, and Amb a 1-specific IgE accounted for more than 50% of ragweed pollen-specific IgE in the majority of ragweed pollen-allergic patients. In the study population from the ragweed-endemic area, we found that 18.7%, 4% and 29.3% reacted to nArt v 1, rBet v 1 and rPhl p 1/5b, the marker allergens for genuine sensitization to mugwort, birch, and timothy grass pollen, respectively (Table S1), which corresponded to the SPT data (Table 1). Interestingly, the comparison of extract sIgE and nAmb a 1 sIgE, showed that 19% of the patients had a higher amount of nAmb a 1 sIgE compared to the extract (Figure 2). A similar observation has been made earlier for timothy grass pollen extract [19], and may be explained by the fact that in ragweed pollen Amb a 1 represents only a fraction of the proteins and allergens present in the crude extract. Accordingly, the content of Amb a 1 may be lower in the ragweed pollen extract and thus is not sufficient for binding all Amb a 1-specific antibodies. This further indicates the presence of other relevant allergens in ragweed pollen. In fact, for around 40% of the patients in our study cohort, Amb a 1 was responsible for 80% or fewer allergen-specific IgE directed to the whole extract, which again emphasises our previous observation that there may be other important allergens which contribute to the ragweed IgE sensitization profile. Interestingly, IgE reactivity to nAmb a 1 was not significantly higher in patients with more different symptoms or associated with severe symptoms like asthma. Additionally, these findings suggest that, besides Amb a 1, other allergens also play a role in inducing allergy symptoms as previously reported [35]. So far, no significant association could be found in our study between IgE response to pan-allergens and different phenotypes of ragweed allergy, so in the future the complete panel of ragweed allergens needs to be tested.

When testing the frequency of IgE recognition to rAmb a 8, we found that 21.3% of the study cohort was sensitized to this allergen (Table S1). These patients were then tested for IgE reactivity to profilins from mugwort, birch and grass. All (*n* = 32) rAmb a 8-sensitized patients also reacted to rArt v 4 and rPhl p 12 (Table 2). Three Amb a 8-positive patients were negative for rBet v 2, but the specific IgE levels to profilins in these patients were low. It has been suggested that cross-reactivity and co-sensitization can be distinguished by the quantification of allergen-specific IgE levels [37]. In our study, many of the Amb a 8-reactive patients showed comparable IgE levels towards other profilins, indicating co-sensitization, but we found also several for whom Amb a 8-specific IgE levels were higher than those for other profilins. In contrast to a previous report stating that primary sensitization is usually detected by IgE sensitization to major allergens [38], it seems that one can also consider IgE levels to cross-reactive allergens for the identification of the primary sensitizing allergen source. In our study, all (*n* = 32) profilin-positive patients reacted to nAmb a 1, whereas only twelve patients (37.5%) showed reactivity to rPhl p 1/5b, six to nArt v 1 (18.7%) and three to rBet v 1 (9.3%) (Table S1). More than half (56.2%) of the profilin-positive patients were sensitized only to nAmb a 1 and not to other major allergens tested, indicating that ragweed was the primary sensitizing allergen source for the Amb a 8-sensitized patients (Table S1). This conclusion was supported by ImmunoCAP inhibition experiments. Amb a 8 was able to inhibit IgE binding to all other profilins with a median reduction of ≥92% (Table 3), indicating a high degree of cross-reactivity, whereas Art v 4 inhibited IgE binding to Amb a 8 to a lower extent with a median reduction of 80% (Table 3). These findings again support Amb a 8 as the primary sensitizer to profilin in our study population. We can therefore confirm that the measurement of specific IgE levels to highly cross-reactive allergens can be helpful for the identification of primary sensitization to an allergen source, in addition to positivity for major allergens as has been shown for albumin earlier [39].

IgE cross-reactivity with profilins and other highly cross-reactive allergens is a problem for allergen-extract-based diagnosis because patients will show IgE cross-reactivity and sensitivity to allergen extracts containing the cross-reactive allergens, which will obscure the identification of the genuinely sensitizing allergen source [27]. We evaluated the allergenic activity in mediator release assays. HRBLs sensitized with sera from seven profilin-positive patients and stimulated with rAmb a 8 and rArt v 4 showed that both proteins can induce degranulation (Figure 3). These data suggest that weed profilins play an important role in triggering allergy symptoms in patients sensitized to this pan-allergen. Accordingly, one can expect positive test results when using profilin-containing allergen extracts from different allergen sources for IgE testing, basophil activation testing and SPT.

We also evaluated the ragweed calcium-binding proteins rAmb a 9 and rAmb a 10. We started by measuring the IgE reactivity of the study cohort in ELISA using rAmb a 9, rAmb a 10 and rArt v 5, resulting in IgE reactivity of 16%, 25% and 35%, respectively (Figure 4). These results are comparable with a previous report [25].

CCDs are known to bind IgE of patients who are allergic to venoms, pollen and mites [40]. We investigated the IgE reactivity of CCD within our cohort of ragweed-allergic patients. A total of 13% of our patients were positive for CCD in ImmunoCAP (Table S1), indicating a cross-reactive reaction, but which ragweed allergen is involved in this cross-reactivity remains unknown. However, strong sensitization exclusively to CCD was not encountered within our cohort. For better accuracy of the ragweed allergy diagnosis, this cross-reactivity between CCD and the ragweed allergen needs to be identified and considered in CRD.

The ImmunoCAP platform has proven to be a helpful tool in detecting ragweed and other pollen sensitizations in our study population. The genuine sensitizing sources could be identified in almost all of the patients included in our study cohort (Table S1). One patient appeared to be sensitized to mugwort, and IgE reactivity to ragweed extract could be due to cross-reactivity between Art v 1 and its homologue Amb a 4 in ragweed, but we have not tested for IgE reactivity to the latter allergen and additional other ragweed pollen allergens. Nevertheless, our study together with available information regarding IgE recognition of ragweed allergen molecules allows us to propose a refined algorithm for identifying genuinely ragweed-sensitized allergic patients by molecular diagnosis, which may provide a basis for personalized forms of treatment (e.g., allergen-specific immunotherapy) and prevention (e.g., allergen avoidance).

**Figure 6:**
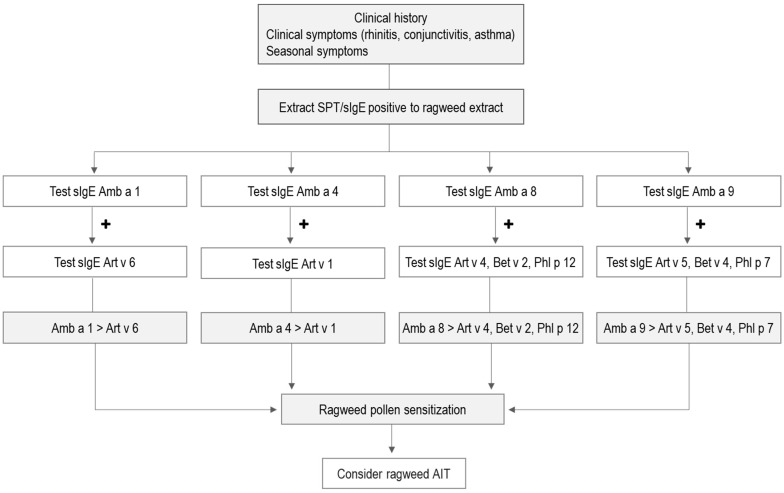
Diagnosis algorithm and decision tree for identification of ragweed pollen allergy by CRD in areas where ragweed, mugwort, birch and timothy grass pollen are found. Patients with symptoms of allergic rhinitis during the ragweed pollen season and a positive SPT/IgE assay to ragweed pollen extract are further investigated to detect IgE antibodies in serum to Amb a 1, Amb a 4, Amb a 8 and Amb a 9. Additionally, investigation of the IgE antibodies to cross-reactive molecules, homologous to ragweed allergens (i.e., Art v 1, Art v 4, Art v 5, Art v 6, Bet v 2, Bet v 4, Phl p 12 and Phl p 7), are recommended before AIT prescription.

In Figure 6 we display a possible integration of major and minor allergen components in the identification of genuine ragweed sensitization in areas with a high prevalence of ragweed, mugwort, birch and timothy grass pollen. Although Amb a 8 and Amb a 9 are not yet available for molecular diagnosis, our study showed that the inclusion of these pan-allergens in the molecular diagnosis, together with Amb a 1 and Amb a 4, may provide useful information about the patient IgE sensitization profile and further facilitate the prescription of ragweed AIT. Accordingly, we recommend that patients reporting allergy symptoms to ragweed pollen, confirmed positive by SPT or serum IgE testing to ragweed pollen extract, be further investigated for IgE antibodies to molecular components Amb a 1, Amb a 4, Amb a 8 and Amb a 9. Patients positive to either Amb a 1, Amb a 4, Amb a 8 or Amb a 9 should be further investigated for their IgE antibodies to potentially cross-reactive homologous allergens. Patients having higher IgE levels to ragweed allergens compared to their homologues are considered to be genuinely sensitized to ragweed pollen. Although our algorithm is useful in identifying genuine ragweed sensitization, co-sensitization to other pollens cannot be excluded. However, patients who exhibit allergy symptoms upon ragweed pollen exposure and higher IgE levels to ragweed allergens, in the absence of symptoms to other pollen, could consider the prescription of ragweed AIT provided that the immunotherapy induces IgG antibodies against the given allergens. This can be determined by the immunization of animals or by the analysis of sera from patients who had been treated regarding the induction of IgG to the allergens in question by the treatment [41,42].

It should be noted that our algorithm is adequate only for areas where ragweed, mugwort, birch and timothy grass pollen are found. In regions with an abundance of olive or *Parietaria* pollen, this algorithm has to be adapted to also include the relevant pan-allergens from these sources. In this case, it can be stated that Amb a 8-specific IgE should be higher than IgE for other pollen profilins and Amb a 9-specific IgE should be higher than IgE for other pollen CBPs, respectively. We hope our findings will improve the current molecular diagnosis of ragweed allergy and provide useful insight to clinicians regarding the prescription of ragweed AIT in the future.

## 4. Materials and Methods

### 4.1. Patients and Patients’ Sera

Patients (*n* = 150) from an allergy centre in Timisoara, Romania, with a conclusive history of ragweed pollen allergy, allergy symptoms during ragweed pollen season and positive skin prick test and/or IgE testing to ragweed extract were included in this study. Written informed consent was obtained from all the participants of the study. Data concerning clinical symptoms reported by the patients during ragweed pollen season and other sensitization were assessed via a validated questionnaire. Blood samples were collected, centrifuged and stored at −80 °C until use. The study was approved by the local ethics committee of the County Emergency Clinical Hospital “Pius Brinzeu”, Timisoara.

### 4.2. Expression and Purification of the Recombinant Allergens

Synthetic genes coding for Amb a 8.0101, Amb a 9.0101, Amb a 10.0101, Art v 4.0101 and Art v 5.0101 allergens, sequences available on WHO/IUIS Allergen Nomenclature Website [43,44,45,46,47], were synthesized, codon optimized for *Escherichia coli* expression and subcloned into pET27b expression vector (ATG Biosynthetics, Merzhausen, Germany). Recombinant allergens containing a C-terminal hexa-histidine-tag were expressed in *E. coli* BL21-Gold (DE3) (Agilent Technologies, Santa Clara, CA, USA), as described [48]. rAmb a 9, rAmb a 10 and rArt v 5 were purified under native conditions, whereas rAmb a 8 and rArt v 4 were purified under denaturing conditions using Ni-NTA resin affinity columns according to the manufacturer’s protocol (Qiagen, Hilden, Germany). Purified allergens were dialyzed against 10 mM NaH_2_PO_4_ pH 8 (rAmb a 8 and rArt v 4), pH 8,5 (rAmb a 9, rAmb a 10 and rArt v 5), and the final allergen concentration was determined using a PierceTM BCA Protein Assay Kit (Thermo Fisher Scientific, Waltham, MA, USA). Purified allergens were further stored at −20 °C.

### 4.3. Allergen-Specific IgE Reactivity Determined in ELISA

Elisa plates (MaxiSorp, Thermo Fisher Scientific, Waltham, MA, USA) were coated overnight with rAmb a 9, rAmb a 10 and rArt v 5 (5 µg/mL in PBS) at 4 °C. Plates were washed two times with PBS 0.05% (*vol*/*vol*) Tween 20 (PBST) and non-specific binding sites were blocked with PBST containing 3% (*wt*/*vol*) Bovine Serum Albumin (BSA) for 2.5 h at room temperature. Sera from ragweed pollen-allergic patients (*n* = 150) were diluted 1:5 with PBST containing 0.5% (*wt*/*vol*) BSA and incubated overnight at 4 °C. Thereafter, plates were washed 5 times with PBST and bound IgE antibodies were detected with 1:2500-diluted horseradish peroxidase (HRP) labelled goat anti-human IgE antibody (SeraCare, Milford, MA, USA) for 45 min at 37 °C and 45 min at 4 °C. After five times of washing with PBST, the colour development staining solution containing 0.387 g citric acid, 0.412 g Na_2_HPO_4_ × H_2_O, 0.030 g ABTS (2,2-azino-bis (3-ethylbenzthiazoline-6-sulfonic acid), diammonium salt) (Sigma Aldrich, St. Louis, MO, USA) and 3 µL H_2_O_2_ in 30mL ddH_2_O was added on the plate (100 µL/well). Optical densities (OD) were measured at 405 nm with reference at 490 nm using a microplate reader (Infinite M200 PRO, Tecan, Männerdorf, Switzerland). All determinations were performed in duplicate. Results represent mean OD values and cut-off values were defined as mean OD plus 3 times the standard deviation of 4 sera from non-allergic patients. Table S1 shows patients’ OD values from which the cut-off value for each allergen was previously subtracted; negative values were set to zero.

### 4.4. Quantification of Specific IgE Levels Using ImmunoCAP

SIgE reactivity measurements were performed using the fluoro-enzyme immunoassay-based ImmunoCAP Phadia 100/250 platform (Thermo Fisher Scientific/Phadia, Uppsala, Sweden) according to the manufacturer’s guidelines. Positive values were considered ≥0.35 kUA/L, as indicated by the manufacturer. Patients were tested for their sIgE to ragweed pollen extract (w1 common ragweed, ImmunoCAP, Thermo Fisher Scientific/Phadia, Uppsala Sweden), nAmb a 1 (w230), nArt v 1 (w231), rBet v 1 (t215), rPhl p 1/5b (g213), rBet v 2 (t216) and rPhl p 12 (g212). Further, patients were tested with Streptavidin ImmunoCAPs (o212 ImmunoCAP, Thermo Fisher Scientific/Phadia) containing rAmb a 8, rArt v 4 and ProGlycAn CCD (Hämosan, Ilz, Austria), as described [49]. Allergens and CCD were dialyzed against a carbonate buffer (0.1 M NaHCO3, 1 M NaCl) and incubated with a fivefold molar excess of biotin (Biotinamidohexanoyl-6-aminohexanoic acid N- hydroxysuccinimide ester, Sigma, St. Louis, MO, USA) for 3 h. Excess biotin was removed by dialyzing against DPBS. Prewashed Streptavidin ImmunoCAPs were loaded with 50 μL of the biotinylated allergens and ProGlycAn (100 μg/mL) and incubated for 30 min at room temperature. IgE reactivity to rAmb a 8, rArt v 4 and ProGlycAn P was determined according to the manufacturer’s guidelines.

### 4.5. Evaluation of Amb a 1 Specific IgE Proportion Corresponding to Ragweed Extract sIgE

The proportion of nAmb a 1 sIgE corresponding to the extract sIgE was evaluated. For each patient, the value of nAmb a 1 sIgE (kUA/L) was divided by the value of ragweed pollen extract sIgE to show how much of the patients’ response to the extract is due to nAmb a 1. The percentage was calculated and used to display the different proportions of nAmb a 1 sIgE in the extract sIgE.

### 4.6. ImmunoCAP Inhibition Experiments

Fifty microliters of serum were preincubated with fifty microliters of the inhibitor allergen rAmb a 8 or rArt v 4 in PBS buffer (final concentration of 100 µg/mL). The mix was incubated overnight at 4 °C and ImmunoCAP testing was then performed with these sera according to the manufacturer’s guidelines. IgE reactivity to rPhl p 12, rBet v 2, rArt v 4 and rAmb a 8 was assessed with rAmb a 8-inhibited sera and IgE reactivity to rAmb a 8 was assessed with rArt v 4-inhibited sera.

### 4.7. β-Hexosaminidase Release Assay Using RBL RS-ATL8

Rat basophil leukemia (RBL) RS-ATL8 cell line transfected with the human high-affinity IgE receptor (FcεRIH) [50] was kindly provided by Dr. Ryosuke Nakamura (Tokyo, Japan). Cells were plated (1.5 × 10^5^ cells/well) on 96-well sterile tissue culture plates (Corning, New York, NY, USA) added with cell culture medium MEM supplemented with 10% FCS, 0.2 mM L-Glutamine, 100 U/mL Pen/Strep 0.2 mg/mL geneticin (Gibco, Thermo Fischer Scientific, Waltham, MA, USA) and 0.2 mg/mL hygromycin B (Invitrogen, Thermo Fischer Scientific, Waltham, MA, USA) and loaded with 1:10 diluted serum, previously heat-inactivated 25 min at 56 °C [51] and then incubated overnight at 37 °C, 5% CO_2_. For this experiment, sera from seven patients positive in ImmunoCAP to ragweed profilin within our study cohort were used. After washing with Tyrod Buffer containing Tyrode’s Salts (Sigma-Aldrich, Vienna, Austria) and 1.0 g/L NaHCO_3_, degranulation of RBL cells was induced upon stimulation with 6 different concentrations of rAmb a 8 and rArt v 4 (0.01–1000 ng/mL). nAmb a 1.01, produced as described [52], was used for control purposes. Buffer without allergens was used as the negative control. Results are shown as the percentage of total ß-hexosaminidase release. All measurements were performed in triplicate and represent means ± SDs.

### 4.8. Statistical Analysis

Statistical analysis was performed using SPSS version 20.0 (SPSS Inc., Chicago, IL, USA). SIgE values obtained in ImmunoCAP were compared using statistical tests. The Shapiro–Wilk test was used to verify the distribution of the population. Nonparametric data of two related samples were compared with the Wilcoxon signed-rank test, whereas independent samples were compared with the Kruskal–Wallis one-way analysis of variance test. *p* < 0.05 levels were regarded as statistically significant.

## 5. Conclusions

Our study showed that the vast majority of our study population reporting ragweed seasonal allergy symptoms were genuinely ragweed-sensitized. The sensitization prevalence to other allergen sources appeared higher due to the profilin cross-reactivity. Quantitation of specific IgE has proven to be helpful when distinguishing between the primary sensitizer and cross-reactive allergens.

We found Amb a 8 to be the primary profilin-sensitizer in ragweed-allergic patients in the absence of other pollen sensitizations. Amb a 8 seems to be a clinically relevant allergen. Therefore, Amb a 8 needs to be considered for component-resolved diagnosis and the development of molecular-based allergen-specific immunotherapy.

## Figures and Tables

**Table 1:** Sensitizations and prevalence of symptoms in ragweed-allergic patients.

Patients, no.	150
Sex male/female no. (%)	100/50 (66/34)
Age range (median)	18–61 (35)
**Prevalence of symptoms (%)**	
Rhinitis	100
Conjunctivitis	93
Asthma-like symptoms	66
Skin symptoms	26
**Other sensitizations (%)**	
House dust mites	37
Grass pollen	34
Mugwort pollen	30
Tree pollen	20
Fungi	19
Dog	15
Cat	15

**Table 2:** ImmunoCAP measurement of sIgE (kUA/L) against profilins rAmb a 8 (ragweed), rArt v 4 (mugwort), rBet v 2 (birch) and rPhl p 12 (timothy grass) (*n* = 32).

Pat. No.	Specific IgE (kUA/L)			
	rAmb a 8	rArt v 4	rBet v 2	rPhl p 12
#3	6.76	3.94	2.25	2.48
#5	1.36	1.85	1.68	1.21
#6	0.47	0.5	0.38	0.5
#7	4.27	5.43	4.69	3.4
#18	0.36	0.35	0.81	0.53
#28	5.14	5.54	6.01	4.48
#31	0.54	0.59	0.49	0.45
#37	2.22	2.95	3.63	2.77
#39	0.5	0.67	0.71	0.4
#41	22.6	36.8	41.2	20
#46	13.7	11.1	6.88	8.38
#52	0.54	0.82	0.88	0.41
#54	1.21	1.15	1.14	0.93
#58	6.19	7.63	6.79	4.26
#62	3.47	3.24	2.33	2.31
#63	78.4	40.5	33.3	43.2
#70	0.63	0.65	0.6	0.71
#71	0.38	0.49	0.11	0.44
#72	38.7	55.4	37.5	34.9
#76	14.7	16.7	14	15.4
#83	12	16.2	12.8	10.1
#103	15.1	11.8	11	9.01
#106	0.91	1.1	0.05	0.52
#108	0.4	0.5	0.34	0.38
#113	3.3	4.84	3.73	2.57
#115	4.71	5.83	4.98	3.5
#117	6.26	7.07	6.03	5.25
#118	0.84	1.04	0.51	0.75
#121	0.72	0.9	0.63	0.67
#132	3.68	4.31	3.32	3.09
#143	1.46	1.68	1.5	1.19
#147	1.34	1.81	1.22	1.2
Median	2.76	3.10	2.29	2.40

Values < 0.35 kUA/L are below the cut-off proposed by the manufacturer (grey).

**Table 3:** Inhibition of allergic patients’ IgE binding to profilins rAmb a 8 (ragweed), rArt v 4 (mugwort), rBet v 2 (birch) and rPhl p 12 (timothy grass) by serum pre-incubation with Amb a 8 and inhibition of IgE binding to Amb a 8 by Art v 4.

Pat. No.	rArt v 4 sIgE			rBet v 2 sIgE			rPhl p 12 sIgE			rAmb a 8 sIgE				
	Non-inhib.	Inhib. with Amb a 8	%	Non-inhib.	Inhib. with Amb a 8	%	Non-inhib.	Inhib. with Amb a 8	%	Non-inhib.	Inhib. with Amb a 8	%	Inhib. with Art v 4	%
#3	3.94	0.04	99	2.25	0.11	95	2.48	0.07	97	6.76	0.21	97	2.76	59
#5	1.85	0.00	100	1.68	0.05	97	1.21	0.04	97	1.36	0.06	96	0.29	79
#6	0.5	0.00	100	0.38	0.01	97	0.5	0.01	97	0.47	0.02	96	0.11	77
#7	5.43	0.05	99	4.69	0.37	92	3.4	0.18	95	4.27	0.12	97	0.30	93
#18	0.35	0.00	100	0.81	0.20	75	0.53	0.09	82	0.36	0.03	92	0.01	97
#28	5.54	0.00	100	6.01	1.74	71	4.48	0.74	83	5.14	0.79	85	0.96	81
#31	0.59	0.00	100	0.49	0.00	100	0.45	0.01	99	0.54	0.00	100	0.04	93
#37	2.95	0.00	100	3.63	0.42	88	2.77	0.14	95	2.22	0.06	97	0.25	89
#39	0.67	0.00	100	0.71	0.07	90	0.4	0.05	87	0.5	0.03	94	0.04	92
#41	36.8	0.03	100	41.2	11.59	72	20	3.67	82	22.6	4.30	81	4.33	81
#46	11.1	0.25	98	6.88	0.29	96	8.38	0.12	99	13.7	0.50	96	4.45	68
#52	0.82	0.01	99	0.88	0.14	84	0.41	0.03	93	0.54	0.02	96	0.13	76
#54	1.15	0.00	100	1.14	0.05	96	0.93	0.02	98	1.21	0.01	99	0.30	75
#58	7.63	0.68	91	6.79	1.24	82	4.26	0.44	90	6.19	0.37	94	0.90	85
#62	3.24	0.21	94	2.33	0.32	86	2.31	0.19	92	3.47	0.26	93	0.71	80
#63	40.5	0.18	100	33.3	0.48	99	43.2	0.87	98	78.4	3.21	96	25.78	67
#70	0.65	0.00	100	0.63	0.12	81	0.71	0.11	85	0.63	0.10	84	0.08	87
#72	55.4	0.09	100	37.5	3.15	92	34.9	1.11	97	38.7	2.12	95	7.18	81
#76	16.7	0.01	100	14	0.11	99	15.4	0.09	99	14.7	0.36	98	2.63	82
#83	16.2	0.01	100	12.8	1.69	87	10.1	0.78	92	12	0.94	92	2.38	80
#103	11.8	0.14	99	11	0.21	98	9.01	0.22	98	15.1	0.46	97	5.33	65
#113	4.84	0.63	87	3.73	0.60	84	2.57	0.34	87	3.3	0.47	86	0.34	90
#115	5.83	0.00	100	4.98	0.18	96	3.5	0.06	98	4.71	0.13	97	1.31	72
#117	7.07	0.06	99	6.03	0.08	99	5.25	0.06	99	6.26	0.18	97	1.69	73
#118	1.04	0.01	99	0.51	0.00	100	0.75	0.01	99	0.84	0.02	98	0.16	81
#121	0.9	0.00	100	0.63	0.01	99	0.67	0.02	97	0.72	0.02	97	0.28	61
#132	4.31	0.18	96	3.32	0.28	92	3.09	0.10	97	3.68	0.25	93	0.98	73
#143	1.68	0.00	100	1.5	0.08	94	1.19	0.05	96	1.46	0.09	94	0.33	77
#147	1.81	0.00	100	1.22	0.10	92	1.2	0.09	92	1.34	0.06	95	0.11	92
Med.	3.94	0.01	100	3.32	0.18	92	2.57	0.09	97	3.47	0.13	96	0.34	80

Med = median; non-inhib. = non-inhibited; inhib. = inhibited. Values < 0.35 kUA/L are negative (grey).

## Data Availability

Not applicable.

## Supplementary Materials

The following supporting information can be downloaded at: https://www.mdpi.com/article/10.3390/ijms24044040/s1.

## References

[1] Valenta R., Karaulov A., Niederberger V., Gattinger P., van Hage M., Flicker S., Linhart B., Campana R., Focke-Tejkl M., Curin M. (2018). Molecular Aspects of Allergens and Allergy. Adv. Immunol..

[2] Payne W.W. (1964). A Re-Evaluation of the Genus Ambrosia (Compositae). J. Arnold Arbor..

[3] Kiss L., Béres I. (2006). Anthropogenic Factors behind the Recent Population Expansion of Common Ragweed (*Ambrosia Artemisiifolia* L.) in Eastern Europe: Is There a Correlation with Political Transitions?. J. Biogeogr..

[4] Xu H., Qiang S., Han Z., Guo J., Huang Z., Sun H., He S., Ding H., Wu H., Wan F. (2006). The Status and Causes of Alien Species Invasion in China. Biodivers. Conserv..

[5] McFadyen R.E. (1984). Annual Ragweed in Queensland. Proceedings of the Seventh Australian Weeds Conference.

[6] Essl F., Biró K., Brandes D., Broennimann O., Bullock J.M., Chapman D.S., Chauvel B., Dullinger S., Fumanal B., Guisan A. (2015). Biological Flora of the British Isles: Ambrosia Artemisiifolia. J. Ecol..

[7] Bassett I.J., Crompton C.W. (1975). The biology of Canadian weeds: 11. *Ambrosia Artemisiifolia* L. and *A. Psilostachya* DC. Can. J. Plant Sci..

[8] Siroux V., Lupinek C., Resch Y., Curin M., Just J., Keil T., Kiss R., Carlsen K.L., Melen E., Nadif R. (2017). Specific IgE and IgG Measured by the MeDALL Allergen-Chip Depend on Allergen and Route of Exposure: The EGEA Study. J. Allergy Clin. Immunol..

[9] Skjøth C.A., Smith M., Šikoparija B., Stach A., Myszkowska D., Kasprzyk I., Radišić P., Stjepanović B., Hrga I., Apatini D. (2010). A Method for Producing Airborne Pollen Source Inventories: An Example of Ambrosia (Ragweed) on the Pannonian Plain. Agric. For. Meteorol..

[10] Rasmussen K., Thyrring J., Muscarella R., Borchsenius F. (2017). Climate-Change-Induced Range Shifts of Three Allergenic Ragweeds (*Ambrosia* L.) in Europe and Their Potential Impact on Human Health. PeerJ.

[11] Lu S., Luo X., Han L., Yang J., Jin J., Yang J. (2022). Genetic Patterns Reveal Differences between the Invasion Processes of Common Ragweed in Urban and Non-Urban Ecosystems. Glob. Ecol. Conserv..

[12] Dahl Å., Strandhede S.-O., Wihl J.-Å. (1999). Ragweed–an Allergy Risk in Sweden?. Aerobiologia.

[13] Heinzerling L., Frew A.J., Bindslev-Jensen C., Bonini S., Bousquet J., Bresciani M., Carlsen K., Van Cauwenberge P., Darsow U., Fokkens W.J. (2005). Standard Skin Prick Testing and Sensitization to Inhalant Allergens across Europe–a Survey from the GA2LEN Network. Allergy.

[14] Valenta R., Karaulov A., Niederberger V., Zhernov Y., Elisyutina O., Campana R., Focke-Tejkl M., Curin M., Namazova-Baranova L., Wang J.Y. (2018). Allergen Extracts for In Vivo Diagnosis and Treatment of Allergy: Is There a Future?. J. Allergy Clin. Immunol. Pract..

[15] Santos A.F., Brough H.A. (2017). Making the Most of in Vitro Tests to Diagnose Food Allergy. J. Allergy Clin. Immunol. Pract..

[16] Ansotegui I.J., Melioli G., Canonica G.W., Caraballo L., Villa E., Ebisawa M., Passalacqua G., Savi E., Ebo D., Gómez R.M. (2020). IgE Allergy Diagnostics and Other Relevant Tests in Allergy, a World Allergy Organization Position Paper. World Allergy Organ. J..

[17] Focke M., Marth K., Valenta R. (2009). Molecular Composition and Biological Activity of Commercial Birch Pollen Allergen Extracts. Eur. J. Clin. Investig..

[18] Matricardi P.M., Kleine-Tebbe J., Hoffmann H.J., Valenta R., Hilger C., Hofmaier S., Aalberse R.C., Agache I., Asero R., Ballmer-Weber B. (2016). EAACI Molecular Allergology User’s Guide. Pediatr. Allergy Immunol..

[19] Valenta R., Lidholm J., Niederberger V., Hayek B., Kraft D., Grönlund H. (1999). The Recombinant Allergen-Based Concept of Component-Resolved Diagnostics and Immunotherapy (CRD and CRIT). Clin. Exp. Allergy.

[20] WHO/IUIS Allergen Nomenclature Sub-Committee. www.allergen.org.

[21] Pablos I., Eichhorn S., Machado Y., Briza P., Neunkirchner A., Jahn-Schmid B., Wildner S., Soh W.T., Ebner C., Park J. (2017). Distinct Epitope Structures of Defensin-like Proteins Linked to Proline-rich Regions Give Rise to Differences in Their Allergenic Activity. Allergy.

[22] Van Hage M., Hamsten C., Valenta R. (2017). ImmunoCAP Assays: Pros and Cons in Allergology. J. Allergy Clin. Immunol..

[23] Van Hage M., Schmid-Grendelmeier P., Skevaki C., Plebani M., Canonica W., Kleine-Tebbe J., Nystrand M., Jafari-Mamaghani M., Jakob T. (2017). Performance Evaluation of ImmunoCAP^®^ ISAC 112: A Multi-Site Study. Clin. Chem. Lab. Med..

[24] Buzzulini F., Da Re M., Scala E., Martelli P., Conte M., Brusca I., Villalta D. (2019). Evaluation of a New Multiplex Assay for Allergy Diagnosis. Clin. Chim. Acta.

[25] Wopfner N., Gruber P., Wallner M., Briza P., Ebner C., Mari A., Richter K., Vogel L., Ferreira F. (2008). Molecular and Immunological Characterization of Novel Weed Pollen Pan-allergens. Allergy.

[26] Valenta R., Duchene M., Ebner C., Valent P., Sillaber C., Deviller P., Ferreira F., Tejkl M., Edelmann H., Kraft D. (1992). Profilins Constitute a Novel Family of Functional Plant Pan-Allergens. J. Exp. Med..

[27] Kazemi-Shirazi L., Niederberger V., Linhart B., Lidholm J., Kraft D., Valenta R. (2002). Recombinant Marker Allergens: Diagnostic Gatekeepers for the Treatment of Allergy. Int. Arch. Allergy Immunol..

[28] Ebo D.G., Hagendorens M.M., Bridts C.H., De Clerck L.S., Stevens W.J. (2004). Sensitization to Cross-reactive Carbohydrate Determinants and the Ubiquitous Protein Profilin: Mimickers of Allergy. Clin. Exp. Allergy.

[29] Hemmer W., Focke M., Kolarich D., Wilson I.B., Altmann F., Wohrl S., Gotz M., Jarisch R. (2001). Antibody Binding to Venom Carbohydrates Is a Frequent Cause for Double Positivity to Honeybee and Yellow Jacket Venom in Patients with Stinging-Insect Allergy. J. Allergy Clin. Immunol..

[30] Ianovici N., Panaitescu C.B., Brudiu I. (2013). Analysis of Airborne Allergenic Pollen Spectrum for 2009 in Timişoara, Romania. Aerobiologia.

[31] Chen K.-W., Marusciac L., Tamas P.T., Valenta R., Panaitescu C. (2018). Ragweed Pollen Allergy: Burden, Characteristics, and Management of an Imported Allergen Source in Europe. Int. Arch. Allergy Immunol..

[32] Laaidi M., Laaidi K., Besancenot J.-P., Thibaudon M. (2003). Ragweed in France: An Invasive Plant and Its Allergenic Pollen. Ann. Allergy, Asthma Immunol..

[33] Ianovici N., Juhasz M., Kofol-Seliger A., Sikoparija B. (2009). Comparative Analysis of Some Vernal Pollen Concentrations in Timisoara (Romania), Szeged (Hungary), Novi Sad (Serbia) and Ljubljana (Slovenia). Not. Bot. Horti Agrobot. Cluj-Napoca.

[34] Norman P.S., Winkenwerder W.L., Lichtenstein L.M. (1968). Immunotherapy of Hay Fever with Ragweed Antigen E: Comparisons with Whole Pollen Extract and Placebos. J. Allergy.

[35] Buzan M., Zbîrcea L., Gattinger P., Babaev E., Stolz F., Valenta R., Păunescu V., Panaitescu C., Chen K. (2022). Complex IgE Sensitization Patterns in Ragweed Allergic Patients: Implications for Diagnosis and Specific Immunotherapy. Clin. Transl. Allergy.

[36] Haidar L., Tamas T.-P., Stolz F., Petrisor Patrascu R.F., Chen K.-W., Panaitescu C. (2021). Symptom Patterns and Comparison of Diagnostic Methods in Ragweed Pollen Allergy. Exp. Ther. Med..

[37] Kleine-Tebbe J., Jappe U. (2017). Molecular Allergy Diagnostic Tests: Development and Relevance in Clinical Practice. Allergol. Sel..

[38] McKenna O.E., Asam C., Araujo G.R., Roulias A., Goulart L.R., Ferreira F. (2016). How Relevant Is Panallergen Sensitization in the Development of Allergies?. Pediatr. Allergy Immunol..

[39] Curin M., Swoboda I., Wollmann E., Lupinek C., Spitzauer S., van Hage M., Valenta R. (2014). Microarrayed Dog, Cat, and Horse Allergens Show Weak Correlation between Allergen-Specific IgE and IgG Responses⋆. J. Allergy Clin. Immunol..

[40] Gattinger P., Mittermann I., Lupinek C., Hofer G., Keller W., Stojkovic U.B., Korosec P., Koessler C., Novak N., Valenta R. (2019). Recombinant Glycoproteins Resembling Carbohydrate-Specific IgE Epitopes from Plants, Venoms and Mites. EBioMedicine.

[41] Rodríguez-Domínguez A., Berings M., Rohrbach A., Huang H.-J., Curin M., Gevaert P., Matricardi P.M., Valenta R., Vrtala S. (2020). Molecular Profiling of Allergen-Specific Antibody Responses May Enhance Success of Specific Immunotherapy. J. Allergy Clin. Immunol..

[42] Chen K.-W., Zieglmayer P., Zieglmayer R., Lemell P., Horak F., Bunu C.P., Valenta R., Vrtala S. (2019). Selection of House Dust Mite–Allergic Patients by Molecular Diagnosis May Enhance Success of Specific Immunotherapy. J. Allergy Clin. Immunol..

[43] WHO/IUIS Allergen Nomenclature Sub-Committee Amb a 8. http://allergen.org/viewallergen.php?aid=39.

[44] WHO/IUIS Allergen Nomenclature Sub-Committee Amb a 9. http://allergen.org/viewallergen.php?aid=40.

[45] WHO/IUIS Allergen Nomenclature Sub-Committee Amb a 10. http://allergen.org/viewallergen.php?aid=33.

[46] WHO/IUIS Allergen Nomenclature Sub-Committee Art v 4. http://allergen.org/viewallergen.php?aid=92.

[47] WHO/IUIS Allergen Nomenclature Sub-Committee Art v 5. http://allergen.org/viewallergen.php?aid=93.

[48] Chen K.-W., Fuchs G., Sonneck K., Gieras A., Swoboda I., Douladiris N., Linhart B., Jankovic M., Pavkov T., Keller W. (2008). Reduction of the in Vivo Allergenicity of Der p 2, the Major House-Dust Mite Allergen, by Genetic Engineering. Mol. Immunol..

[49] Weghofer M., Dall’Antonia Y., Grote M., Stöcklinger A., Kneidinger M., Balic N., Krauth M., Fernández-Caldas E., Thomas W.R., van Hage M. (2008). Characterization of Der p 21, a New Important Allergen Derived from the Gut of House Dust Mites. Allergy.

[50] Nakamura R., Uchida Y., Higuchi M., Nakamura R., Tsuge I., Urisu A., Teshima R. (2010). A Convenient and Sensitive Allergy Test: IgE Crosslinking-Induced Luciferase Expression in Cultured Mast Cells. Allergy Eur. J. Allergy Clin. Immunol..

[51] Takagi K., Nakamura R., Teshima R., Sawada J.I. (2003). Application of Human FcεRI α-Chain-Transfected RBL-2H3 Cells for Estimation of Active Serum IgE. Biol. Pharm. Bull..

[52] Wolf M., Twaroch T.E., Huber S., Reithofer M., Steiner M., Aglas L., Hauser M., Aloisi I., Asam C., Hofer H. (2017). Amb a 1 Isoforms: Unequal Siblings with Distinct Immunological Features. Allergy.

